# High-Quality Genome Sequence of *Bacillus anthracis* Strain 14RA5914 Isolated during an Outbreak in Germany in 2014

**DOI:** 10.1128/genomeA.01002-17

**Published:** 2017-10-05

**Authors:** Mandy C. Elschner, Anne Busch, Annette Schliephake, Wolfgang Gaede, Eric Zuchantke, Herbert Tomaso

**Affiliations:** a Friedrich-Loeffler-Institut, Federal Research Institute for Animal Health, Institute of Bacterial Infections and Zoonoses, Jena, Germany; b Department of Veterinary Medicine, State Institute for Consumer Protection of Saxony-Anhalt, Stendal, Germany

## Abstract

*Bacillus anthracis* is a zoonotic agent causing anthrax, a notifiable disease in animals. The last anthrax outbreak among cattle in Germany occurred in April 2014 in Saxony-Anhalt. Here we report a high-quality genome sequence of the *Bacillus anthracis* strain 14RA5914 Dobichau isolated from the spleen of a dead cow.

## GENOME ANNOUNCEMENT

*Bacillus anthracis* causes the zoonotic disease anthrax. Only three natural outbreaks were reported in the past 10 years (1–3). The last outbreak occurred in 2014 in a cattle population in the federal state of Saxony-Anhalt and resulted in 4 dead animals. One animal was diagnostically analyzed, and *B. anthracis* was isolated from a broad range of tissues, including spleen, kidney, synovial fluid, lung, trachea, uterus, intestinal lymph nodes, and colon. The presence of *B. anthracis*-specific genomic DNA and both virulence plasmids pX01 and pX02 was confirmed by real-time PCR assays (4). *B. anthracis* strain 14RA5914 Dobichau was isolated from the spleen. DNA was extracted from bacterial cells that were cultivated in cell culture flasks for 24 h. Bacterial cells were pelleted by centrifugation (3,600 × *g*) (Hettich Rotana 460R, HET5624) and washed with deionized water. DNA purification was performed according to the manufacturer’s instructions using a Genomic-tip 100/G kit and a genomic DNA buffer set (both from Qiagen, Hilden, Germany) including lysozyme, RNase, and proteinase K treatment. Genome sequencing was carried out by use of single-molecule real-time (SMRT) DNA sequencing (5) at GATC Biotech (Germany) using a PacBio RSII sequencer. Genome assembly was done using the Hierarchical Genome Assembly Process algorithm version 3 (HGAP 3) (6) implemented in PacBio SMRT portal v2.3.0. The HGAP 3 assembly generated three contigs representing the genome of *B. anthracis* 14RA5914. The largest contig corresponded to the chromosome and the two smaller contigs to plasmid sequences. For the circularization of contigs, Circlator (Sanger Institute, United Kingdom) was used (7). Finally, the circular contigs were polished with the RS_Resequencing.1 protocol in SMRT portal v2.3.0 and visualized using the SMRT View tool (PacBio). The chromosome has 5,245,242 bp (average coverage of 101×), plasmid pX01 has 198,129 bp (average coverage of 207×), and plasmid pX02 has 103,124 bp (average coverage of 89×), with a mean GC content of 35.4%. The average coverage indicates that plasmid pX01 is represented twice per cell, while plasmid pX02 is represented just once per cell. These findings are in concordance with those for other published *B. anthracis* strains (8). The assembled contigs were submitted to the PROKKA annotation pipeline (NCBI Prokaryotic Genome Annotation Pipeline), resulting in a total of 5,546,495 bp, 33 rRNAs, 6,059 genes, 5,801 coding sequences (CDS), 126 miscellaneous RNAs, 95 tRNAs, and 1 transfer-messenger RNA (tmRNA). The chromosome (5,245,242 bp) contained 33 rRNAs, 5,739 genes, 5,489 CDS, 121 miscellaneous RNAs, 95 tRNAs, and 1 tmRNA. The plasmid pX01 (with 198,129 bp) contained 218 CDS, 220 genes, and 2 miscellaneous RNAs. The plasmid pX02 (103,124 bp) included 117 CDS, 120 genes, and 3 miscellaneous RNAs.

A comparison to the draft genome of a strain of the geographically nearest outbreak of *B. anthracis* in Stendal (3) indicated that *B. anthracis* 14RA5914 contained a larger estimated chromosomal size of 5,227,419 bp, more putative coding sequences (5,639), and more RNA sequences. However, a different sequencing technology and assembly and annotation tools were used, so these observations have to be interpreted with caution.

### Accession number(s).

This whole-genome shotgun project has been deposited at DDBJ/EMBL/GenBank under the accession numbers CP023002 (pX01), CP023003 (pX02), and CP023001 (chromosome). The versions described in this paper are the first versions.
